# Polarity stimulation effects on brainstem auditory evoked potentials

**DOI:** 10.1016/S1808-8694(15)31383-5

**Published:** 2015-10-17

**Authors:** Janaina Patricio de Lima, Kátia de Freitas Alvarenga, Tábata Pierini Foelkel, Camila Zotelli Monteiro, Raquel Sampaio Agostinho

**Affiliations:** 1 Speech therapist; 2 Doctor, Livre-Docente (habilitation) professor; 3 Speech therapist; 4 Speech therapist; 5 Speech therapist. Universidade de São Paulo- Faculdade de Odontologia de Bauru - Departamento de Fonoaudiologia (Speech Therapy Department)

**Keywords:** hearing, evoked potentials, auditory evoked potentials

## Abstract

Brainstem Auditory Evoked Potentials are considered exogenous potentials, that is, the responses obtained are highly dependent upon the characteristic of the stimulus used to evoke them.

**Aim:**

To investigate the influence of the click stimulus polarity in the study of Brainstem Evoked Response Audiometry (BERA) at different intensities, using insertion-canal earphones.

**Type of Study:**

Clinical.

**Materials and methods:**

33 individuals, aged between 18 and 28, with no auditory alteration were submitted to BERA testing, with click stimulus on the rarefaction, condensation and alternate polarities, in different intensities.

**Results:**

The absolute latencies of the V wave proved to be lower in the rarefaction polarity when compared to the others and, at 80 dBnHL, there was a significant difference between rarefaction and the other polarities for interpeak latencies III-V and I-V. There was a high correlation between the condensation and alternating polarities for absolute and interpeak latencies at 80 dBnHL.

**Conclusion:**

the click stimulus polarity has a significant influence on BERA. In the routine use of the TDH 39 earphone, with alternating polarity, we suggest that condensation polarity is more adequate for standardized comparison purposes, due to the higher similarity of the latencies found in this insertion earphone study.

## INTRODUCTION

Brainstem auditory evoked potentials (BAEP) are synchronic discharges of auditory units from the first portion of the auditory nerve to brainstem structures in response to a given stimulus; they may be characterized by a sequence of seven waves (I, II, III, IV, V, VI and VII) that occur within the initial 10 ms of presentation of a strong acoustic stimulus (80 dBnHL).^1^

BAEP are considered as exogenous potentials in which the nature of stimuli directly affects the response. These features include the type, intensity, presentation rate and polarity of the stimulus. Clicks are commonly used stimuli since they may yield abrupt responses with good neural synchronism for yielding BAEP wave components.^2^

There have been debates in the literature about the influence of stimulus polarity and intensity on the latency of the potential being investigated.

Polarity may be of three types: rarefaction (negative polarity), condensation (positive polarity) and alternating (negative/positive polarity). The response of the auditory system to stimuli differs according to the polarity: in rarefaction there is generally an outward movement from the base of the stirrup and an ascending movement in Corti organ structures; in condensation there is an inward initial movement of the stirrup, followed by an inverse movement to that described above; in alternating polarity there is an association between condensation and rarefaction polarities in subsequent presentations.^3^

The latency difference of auditory evoked potentials obtained with rarefaction and condensation has been reported;^3^, ^4^, ^5^, ^6^, ^7^, ^8^, ^9^, ^10^ some authors have reported that rarefaction polarity is used more often in the clinical routine since it is more sensitive for diagnosis compared to condensation polarity.^2^, ^11^ Rarefaction polarity in most subjects generates lower latency potentials and variability not over 0.1 to 0.2 milliseconds in normal hearing subjects.^2^, ^12^

Various factors may explain this difference between rarefaction and condensation polarities, including: auditory sensitivity, middle ear mechanisms, click frequency, electrode position, auditory diseases and high frequency hearing loss.^3^, ^4^, ^5^, ^6^, ^7^, ^8^, ^9^, ^10^

Stimuli at a lower intensity typically increase the latency and reduce the amplitude of the neural response in the latency-intensity function. This latency increase occurs slowly at intensities ranging from 90 to 60 dBnHL; at lower intensities the increase becomes more rapid. However, waves I, III and V are more easily identified at higher frequencies (80 dBnHL in normal subjects); only wave V is seen at 20 dBnHL, which is used to establish the minimal response level. Thus, analysis of the wave V latency-intensity function may provide information about the cause of hearing loss (conductive or sensorineural cochlear/retrocochlear), depending on the response.^13^ Wave V latency may be normal when high intensity stimuli are presented, but may be abnormal when the intensity is decreased (in cochlear cases). At another moment, the wave V latency-intensity function may be completely shifted to the right of normal limits, and there may be conductive and retrocochlear losses.^13^

The purpose of this study was to verify the influence of click polarity absolute and interpeak latencies of BAEP at different intensities, using insertion phones.

## SERIES AND METHOD

The Research Ethics Committee approved this study (process number 24/2005).

The study included 33 normal voluntary subjects, 17 female and 16 male, aged from 18 to 28 years (mean age - 22.82 years), with no history of any risk for auditory alterations, with auditory thresholds not higher than 25 dBNA and type A tympanometric curves.

Pure tone audiometry was done in an acoustic booth using a Madsen model MD622 audiometer, TDH-39 headphones and an MX-41 pad, calibrated according to norms ISO 8253/IEC 645/ISSO 389. Pure tone thresholds at 0.5 to 8 kHz (air conduction) were investigated; a normal threshold was considered as not more than 25 dBNA.

Acoustic immitance testing was done using an Interacoustics Az7 device to discard subjects with altered middle ears. A type A tympanometric curve was considered normal.^14^ An Interacoustics AZ26 digital middle ear analyzer calibrated according to norms ISO 8253/IEC 645/ ISO 389-1991 was also used.

Testing was done in an acoustic booth with subjects comfortably in the supine position and with closed eyes (to avoid ocular movement artifacts) using 3A insertion phones; the individual impedance was below 5kW and the impedance among individuals was lower than 2KW.

Disposable ECG AG/AGCL electrodes were placed according to the 10-20 International System: the active electrode in the Fz position connected to input 1 of channel 1 interlinked to channel 2 by a jumper; reference electrodes were placed in the A1 and A2 positions (left and right earlobes) connected to input 2 of channels 1 and 2, for simultaneous ipsilateral and contralateral recording of BAEP. The ground electrode was placed in the Fpz position.

Rarefaction, condensation and alternating polarity clicks at 80, 60; 40 and 20dBnHL were used, with different polarity sequences. The presentation rate was 21.2 c/sec; 2,000 clicks were promediated, with doubling of responses. The band-pass filter was 100 to 3000 Hz with a 15ms window.

Absolute wave I, III, V latencies and I - III, III - V and I - V interpeaks at 80 dBnHL were measured, as well as the absolute wave V latency at intensities of 60, 40 and 20 dBnHL.

A descriptive statistical analysis (mean and standard deviation) was undertaken. The ANOVA for repeated measurements test was used for comparing the results of absolute and interpeak latency values among polarities, and the Tukey test was used for analyzing the differences found in that comparison. Pearson’s correlation test was used for verifying the correlation among absolute and interpeak latencies of waves I, III and V in different polarities. The significance value was 5% (p=0.05).

## RESULTS

Tables 1 and 2 show the means and standard deviation of absolute and interpeak latencies at different intensities (80, 60, 40 and 20 dBnHL) according to the click polarity (rarefaction, condensation and alternating). Absolute wave I, III and V latencies and wave I-III, III-V and I-V interpeaks at 80 dBnHL were measured, as well as the absolute wave V latency at intensities of 80, 60, 40 and 20 dBnHL.

**Table 1:** Descriptive statistical analysis of absolute wave I, III, V latencies and I - III, III - V and I - V interpeaks at 80 dBnHL and the absolute wave V latency at intensities of 60, 40 and 20 dBnHL, according to click polarities

			Absolute latencies			
	Wave I	Wave III	Wave V	Wave V	Wave V	Wave V
	80	80	80	60	40	20
R	1,68±,1245	3,75±,2104	5,56±,2673	6,08±,2753	6,87±,3730	7,76±,3948
C	1,71±,1289	3,79±,1436	5,70±,2052	6,08±,2753	6,86±,3444	7,91±,3932
A	1,69±,1051	3,81±,1427	5,66±,2322	6,09±,2953	6,89±,3337	7,92±,3818

Key: R- rarefaction; C- condensation; A- alternating

**Table 2:** Descriptive statistical analysis of I-III, III-V and I-V interpeak latencies at 80 dBnHL, according to click polarities

	Interpeak latencies		
	I-III	III-V	I-V
R	2,06±,2131	1,81±,2278	3,87±,2675
C	2,07±,1345	1,91±,1389	3,98±,1965
A	2,12±,1440	1,85±,1789	3,98±,2317

Key: R- rarefaction; C- condensation; A- alternating.

Tables 3 and 4 show the result of the ANOVA test for the comparison of absolute and interpeak latencies at different polarities. The Tukey test was done for further analysis, shown in Table 5.

**Table 3:** Comparison of absolute latencies (ms) obtained in click polarities (rarefaction, condensation and alternating) according to the tested intensities (dBnHL). ANOVA test for repeated measurements.

Absolute latencies						
	I	III	V	V-60	V-40	V-20
p	,18986	,08352	,00005*	,86729	,89296	,02122*

* p=0.05: statistically significant

**Table 4:** Interpeak latencies at 80 dBnHL. ANOVA test for repeated measurements.

	Interpeak latencies		
	I-III	,III-V	I-V

P	,12531	,02029*	,00128*

* p=0.05: statistically significant.

**Table 5:** Analysis of significantly different absolute and interpeak latencies among click polarities. Tukey test.

	III-V (80 dBnHL)	I-V (80 dBnHL)	V (80 dBnHL)	V (20dBnHL)
R × C	,01530*	,00414*	,00016*	,09104
R × A	,44486	,00414*	,00299*	,81964
C × A	,23383	1,0000	,45646	,02252*

Key: R- rarefaction; C- condensation; A- alternating.

* p=0.05: statistically significant.

Pearson’s correlation test showed a significant correlation in the comparison of absolute waves I, III and V latencies at 80 dBnHL in the various polarities: condensation, rarefaction and alternating (p=0.01).

**Chart 1:**
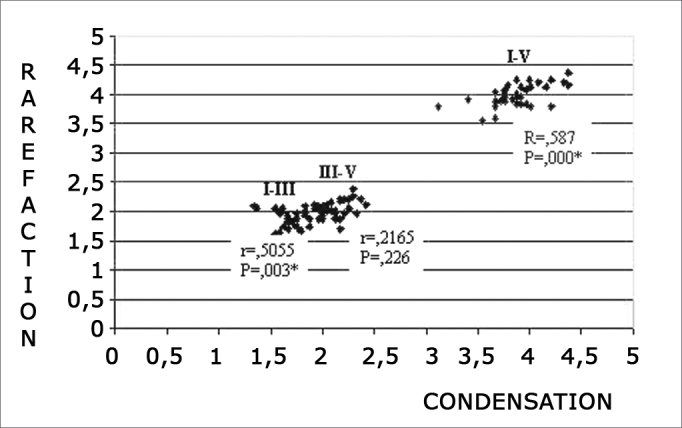
Correlation among I-III, III-V and I-V interpeak latencies in rarefaction and condensation polarities at 80 dBnHL intensity. 0.05: statistically significant. ≤- * p

**Chart 2:**
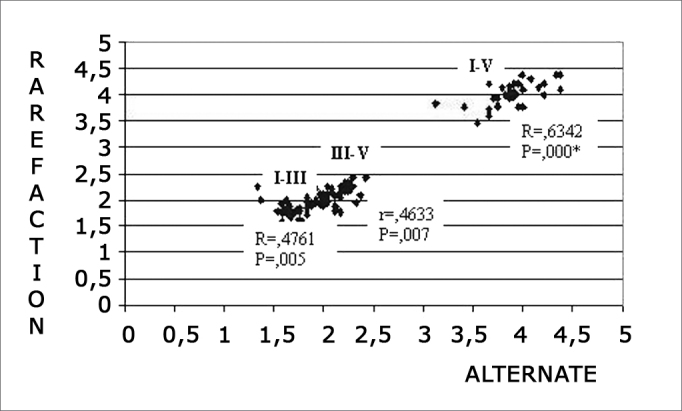
Correlation among I-III, III-V and I-V interpeak latencies in rarefaction and alternating polarities at 80 dBnHL intensity. 0.05: statistically significant. ≤- * p

**Chart 3:**
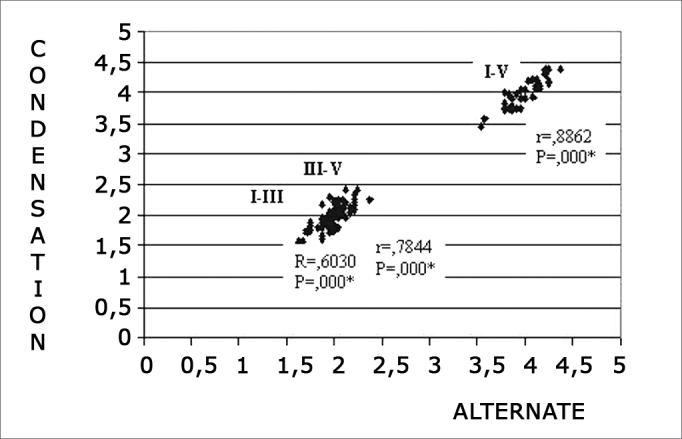
Correlation among I-III, III-V and I-V interpeak latencies in condensation and alternating polarities at 80 dBnHL intensity. 0.05: statistically significant. ≤- * p

Charts 1, 2 and 3 show the result of Pearson’s correlation test for I-III, III-V and I-V interpeak latencies at 80 dBnHL for the various click polarities.

## DISCUSSION

BAEP testing is a widely used procedure in the audiological evaluation. Analysis of wave I, III and V absolute and interpeak latencies and investigation of the electrophysiological threshold using wave V latency-intensity function facilitates the differential diagnosis of cochlear and retrocochlear hearing loss and helps predict the degree of hearing loss when behavioral methods cannot be done.

The nature of the stimulus is a variable that needs to be controlled, as it is an exogenous potential that may significantly affect BAEP recording. Thus, the choice of click polarity has been widely debated in the literature.

There is no clinical consensus about which click polarity is the most appropriate for investigating BAEP. The alternating polarity is the most frequently used one in devices that use the TDH39 headphone, as recording electrical artifacts generated by this transducer may mask wave I (electrical activity generated on the distal portion of the auditory nerve), which increases the difficulty of analysis. Alternating polarity used with signal promediation may decrease the electrical artifact in the recordings, as electrical artifact polarity is similar to that of the stimulus, and is thus cancelled in practice.

The possibility of using insertion 3A headphones in clinical audiology has reduced this problem, since the electrical artifact produced by this transducer is insignificant. Simple polarities (condensation and rarefaction) have become routinely used, mostly to record cochlear microphonism (sensory potential generated in the cochlea); this has helped diagnose auditory neuropathy/auditory dissynchrony, a recently described disorder. An important point is that cochlear microphonism is an alternating current potential, which follows the stimulus polarity; is may thus be cancelled or have its amplitude drastically reduced when using alternating polarity.

Considering that stimulation of Corti organ structures varies according to the polarity,^3^ the recorded electrical activity may have specific features when condensation, rarefaction and alternating polarities are used, as seen in BAEP morphology and latency.^4^, ^15^, ^16^, ^17^, ^18^, ^19^, ^20^, ^21^ Thus, in analyzing the results it is essential to bear in mind that normal absolute and interpeak latencies may vary according to the stimulus polarity. There is, however, no consensus on this in the literature; one study may show that rarefaction is more sensitive and thus superior in clinical practice, while another study states otherwise.^2^

Our findings reveal that mean wave I, III and V absolute and interpeak latency values were generally lower in rarefaction polarity compared to condensation and alternating polarities (Tables 1 and 2). This finding is in agreement with the literature in that reports have shown that rarefaction polarity generates lower latencies compared to condensation polarity.^2^, ^12^, ^20^ No studies were found that took into account the alternating polarity.

At an 80 dBnHL intensity, which is generally used in clinical practice for neurodiagnosis, there was a statistically significant difference between rarefaction polarity and other polarities (condensation and alternating) for the wave V absolute latency and interpeak III-V and I-V latencies (Tables 3, 4 and 5). Therefore, to avoid incorrect diagnoses, normal rarefaction and condensation click response reference values should not be used when analyzing BAEP at 80 dBnHL. On the other hand, if there are no normal alternating polarity reference values, condensation polarity values should be used, since these present no statistically significant differences at 80 dBnHL.

Wave V latency-intensity function analysis may provide relevant information about the type of hearing loss, whether conductive, cochlear or retrocochlear.^13^ Wave V latency analysis obtained at 20 dBnHL differs significantly when condensation or alternating polarities are used (Table 5) and should thus be carefully analyzed.

In normal subjects, absolute wave I, III and V latencies obtained in different polarities and at 80 dBnHL were highly correlated in Pearson’s correlation test. However, interpeak III-V latencies obtained with rarefaction and condensation polarities and interpeak I-III e III-V latencies with rarefaction and alternating polarities were not significantly correlated (Charts 1 and 2), suggesting that a different BAEP (normal or altered) report may exist when done with different polarities. Consistent with the abovementioned data, condensation and alternating polarities were highly correlated for both absolute and interpeak latencies.

Our results suggest that BAEP sensitivity for auditory disorders may vary according to whether rarefaction or condensation polarity clicks are used.^2^

In clinical practice, polarity should be defined so that normal absolute and interpeak latency values are established for making the differential diagnosis of sensorineural hearing loss.

## CONCLUSION

Our study led to the conclusion that the click polarity (condensation, rarefaction and alternating) significantly affects absolute latencies and interpeak latencies of waves I, III and V; the highest difference was in rarefaction polarity. In the routine use of the TDH 39 headphone - with the presentation of the alternating polarity - we suggest using condensation polarity as being more adequate for standardized comparisons, since we found more latency similarity with insertion phones.
